# Factors Associated with Mental Health Literacy Among Undergraduate Health Students in Portuguese Higher Education: The Role of Psychological Well-Being

**DOI:** 10.3390/nursrep16040109

**Published:** 2026-03-27

**Authors:** Ana Isabel Teixeira, Sónia Martins, Sara Lima, Francisca Pinto, Tânia Morgado, Olga Valentim, Hélder Alves

**Affiliations:** 1 Nursing School of Vale do Ave, IPSN-CESPU, 4760-409 Vila Nova de Famalicão, Portugal; 2 RISE-Health, Nursing School of the University of Porto (ESEP), 4200-072 Porto, Portugal; ovalentim@enfermagem.ulisboa.pt; 3 iHealth4Well-Being—Innovation in Health and Well-Being—Research Unit, IPSN-CESPU, 4560-485 Penafiel, Portugal; sonia.martins@ipsn.cespu.pt (S.M.); sara.lima@ipsn.cespu.pt (S.L.); francisca.pinto@ipsn.cespu.pt (F.P.); helder.alves@isssp.pt (H.A.); 4 UCIBIO—Applied Molecular Biosciences Unit, Translational Toxicology Research Laboratory, University Institute of Health Sciences (1H-TOXRUN, IUCS-CESPU), 4585-116 Gandra, Portugal; 5 RISE—Health, Faculty of Medicine, University of Porto, 4200-319 Porto, Portugal; 6 ISSSP—Higher Institute of Social Services of Porto, 4460-362 Porto, Portugal; 7 Nursing School of Tâmega e Sousa, IPSN-CESPU, 4560-485 Penafiel, Portugal; 8 Health Sciences Research Unit: Nursing (UICISA: E), Nursing School of Coimbra (ESEnfC), 3045-043 Coimbra, Portugal; tmorgado@ese.uc.pt; 9 Nursing School of Coimbra, 3004-011 Coimbra, Portugal; 10 Nursing School, University of Lisbon (ESEL), 1600-096 Lisboa, Portugal; 11 Nursing Research, Innovation and Development Centre of Lisbon (CIDNUR), Nursing School of Lisbon (ESEL), 1600-096 Lisboa, Portugal; 12 INESC TEC, 4200-465 Porto, Portugal

**Keywords:** mental health, mental health literacy, psychological well-being, happiness, undergraduate students

## Abstract

**Background**: It is well known that the university period is an important stage for young adults, involving significant academic and psychosocial adjustments. Students with greater Mental Health Literacy (MHL), which is defined as the knowledge, beliefs, and skills individuals have regarding mental health and mental illness, are better able to identify difficulties, seek help, and adopt healthier coping strategies. This study aims to describe the MHL levels of undergraduate health students and identify associated factors related to academic life, mental health and psychological state. **Methods**: A cross-sectional, self-administered, web-based survey was conducted using a non-probability sampling strategy among undergraduate students in health-related degrees at a Portuguese higher-education institution. Data was collected using a general characterization questionnaire and the following instruments: MHL Questionnaire, Academic Life Satisfaction, Subjective Happiness Scale, Psychological Well-Being Scale (PWBS), and Depression Anxiety Stress Scale. Bivariate and linear regression analyses were employed to identify factors associated with MHL. **Results**: A total of 306 students (79% female, mean age = 21.6 years; 59% nursing students) participated. The median MHL score was 70 (range: 30–80). The linear regression model explained 17.5% of the variance in MHL. Higher MHL levels were associated with having the course as a first choice, holding a previous degree, reporting taking psychotropic medication use (which may reflect previous mental health service utilization), and higher levels of psychological well-being. **Conclusions**: This study provides evidence on factors associated with MHL among undergraduate health students, suggesting that higher MHL is associated with greater psychological well-being, highlighting the potential importance of integrating strategies to promote MHL and psychological well-being in health and nursing education. However, these findings should be interpreted with caution due to the single-institution convenience sample, potential self-selection and reporting biases, and cross-sectional design, which limits causal inferences.

## 1. Introduction

The transition to higher education represents a significant milestone in the lives of young adults, marked by various personal, social and academic changes [1]. This transition requires adaptation to new cognitive demands, greater autonomy, changes in family and social ties, and the management of expectations and pressures regarding their professional future [2] as health students [3]. For many students, this phase can be accompanied by emotional instability, academic stress and feelings of insecurity, which contribute to increased vulnerability to mental health (MH) problems such as anxiety and depression and academic dropout [3,4,5].

Recent evidence consistently shows a high burden of MH problems in university students worldwide [4,6,7,8,9]. In Portugal, a cross-sectional study with 3143 university students found that 19.5% reported a diagnosed mental disorder and 23% reported taking psychotropic medication, while 75% reported mild-to-severe anxiety symptoms and 61.2% mild-to-severe depressive symptomatology [10]. These findings underline the relevance of studying MH-related protective factors and help-seeking resources in higher education.

Given the well-documented impact of MH problems on academic outcomes, including poorer academic performance [11] and academic dropout [12,13], these findings highlight the importance of addressing MH in this population. However, a substantial proportion of students do not seek professional help, often due to stigma and lack of knowledge about MH [13].

In this context, Mental Health Literacy (MHL) has emerged as an essential skill, playing a decisive role in identifying, understanding, and managing MH problems in the university environment [14]. MHL is defined as the ability to obtain, understand, and use information to identify, manage, and prevent MH problems [15]. This includes recognizing symptoms of psychological disorders, understanding causes and risk factors, holding less-stigmatizing attitudes, and knowing how to access reliable sources of help and treatment [1]. Greater MHL may facilitate self-compassion and self-care, address negative perceptions and attitudes towards poor MH, and provide clear pathways on how to access MH services when needed [16] to make planned and informed decisions about specialist services in this area.

Taking this into account, it is not surprising that evidence shows that low levels of MHL are closely associated with psychological problems such as anxiety, depression, and symptoms of stress in a broader student population, and that higher levels of MHL are positively correlated with more favorable psychological outcomes, such as well-being, resilience and happiness [3,16,17,18,19,20,21]. However, these constructs are often analyzed separately rather than within a single multidimensional model.

Well-being has been extensively studied in the field of health, with studies demonstrating that it is closely related to MH [21], with undergraduate students with higher levels of well-being reporting greater life satisfaction and lower levels of anxiety and depression [21,22,23]. From a theoretical perspective, well-being has been conceptualized from hedonic and eudaimonic traditions [24,25]. In the present study, psychological well-being (PWB) is framed according to Ryff’s eudaimonic model, which conceptualizes positive psychological functioning as a multidimensional construct [25,26]. Ryff [25], based on the notion of eudaimonic, developed a theoretical model that proposes psychological well-being (PWB) as a multidimensional and eudaimonic approach to well-being, centered on positive psychological functioning and the realization of human potential. The psychological well-being (PWB) model comprises six dimensions [26]: (1) autonomy (self-determination and independence), (2) environmental mastery (a sense of mastery and competence in managing one’s environment), (3) personal growth (a feeling of continued development), (4) purpose in life (having goals in life and a sense of direction), (5) positive relations with others (having warm, satisfying, trusting relationships with others), and (6) self-acceptance (possessing a positive attitude towards the self). Because our analyses use the PWBS total score, this theoretical model is presented as conceptual background rather than as a dimension-by-dimension analytical framework.

From a conceptual standpoint, these aspects of PWB can be relevant to understanding MHL. For instance, greater self-acceptance and personal growth may be associated with greater openness to recognizing psychological difficulties, while environmental mastery and autonomy may be related to individuals’ perceived ability to effectively seek and use mental health information. In this sense, PWB and MHL can be seen as related but distinct constructs that can interact through cognitive, emotional, and social processes involved in recognizing, understanding, and responding to mental health problems.

Given the above, understanding MHL in undergraduate students becomes crucial, as it directly impacts not only students’ well-being but also their academic performance [27]. However, despite the growing recognition of the importance of MHL, it continues to be insufficiently addressed in many educational contexts [4,28].

In a systematic review with meta-analysis, Suwanwong et al. [29] highlighted consistent associations between MHL and various factors, including stigma toward professional help, self-efficacy, attitudes toward help-seeking, social support, positive psychological states, receiving MH training, and psychological distress. However, more robust research methodologies are needed, incorporating longitudinal or repeated assessment of MHL alongside potentially modifiable factors.

In Portugal, a study conducted with 3189 students [30] concluded that students with positive mental health also scored higher in MHL levels and low psychological vulnerability. Another study conducted in the country [10] highlights the urgent need to reflect on pandemic-related changes in academic environments to promote student well-being. It also emphasizes the importance of developing targeted interventions to inform university management decisions based on students’ current mental health status. Furthermore, this study underscores that Portuguese higher-education institutions are not yet adequately prepared to promote students’ mental health.

In this context, the main objective of this study is to describe MHL levels in undergraduate students and the secondary is to identify factors associated with MHL, related to academic life, health and MH history, as well as psychological dimensions, which include PWB and happiness.

Taking these objectives into account, the following research questions are defined: (1) What are the levels of MHL in undergraduate health students? (2) What are the factors associated with higher levels of MHL?

This study assumes as its main hypothesis that MHL is significantly associated with psychological well-being, symptoms of anxiety and depression, and academic-related indicators (e.g., academic satisfaction and perceived performance) among undergraduate health students, with higher MHL expected to relate to more favorable outcomes across these domains.

## 2. Materials and Methods

### 2.1. Design, Participants and Setting

This study had a descriptive cross-sectional design, involving data from the first assessment of the research project: “SMILE—Student mental health literacy for improved academic life satisfaction, happiness, and psychological well-being”, funded by the Department for Research and Innovation of a higher-education institution, located in the north of Portugal, and where this study was carried out. This is a private, non-profit, public utility institution, founded in 1982, dedicated to teaching and research in Health Sciences through its university and polytechnic institutes. These institutes collaborate interdisciplinarily, promoting the exchange of knowledge between different scientific areas and reinforcing the connection between teaching and research. The institution currently has more than 3700 enrolled students.

The main objective of the SMILE research project was to design and implement a brief and tailored intervention focused on improving MHL. This intervention will be tailored to the specific needs of the participants, who will act as active agents in identifying priorities and deciding on the actions to be included. Through workshops, reflective exercises, and interactive activities, students will engage in strategies to improve coping skills, self-awareness, and peer support to empower them to take an active role in promoting their own mental health, fostering skills that can improve both their academic performance and their personal well-being.

Within this framework, an online cross-sectional survey was used to assess the MHL and its potential associated factors among undergraduate health students. The study population comprised all students (aged ≥ 18 years old, both sexes) enrolled in undergraduate health degree programs at this institution. Participants were recruited through a convenience, non-probability sampling approach, and no formal sample size calculation was conducted, as a probability-based sampling strategy was not feasible due to practical constraints. The survey was conducted from September to November 2024.

### 2.2. Data Collection

All undergraduate health students (≥18 years, both sexes) enrolled in health-related degree programs at the institution were invited via an institutional email to participate in this study. The invitation included a link to the survey hosted on the institution’s own online platform, providing detailed information on this study. Participants who gave their free and informed consent by clicking on the online form received a follow-up email with the link to the survey. To enhance understanding, the online platform provided clear instructions for each questionnaire, definitions of key terms, and examples where necessary. Reminder emails were sent at one and two weeks after the initial invitation to increase response rates.

### 2.3. Study Tools

The online survey included a research protocol with a semi-structured questionnaire created for this study. The content of this questionnaire was prepared to gather information about the respondents’ sociodemographic, academic background, and clinical history (e.g., personal and family MH conditions). In addition, this protocol also encompassed the following standardized and previously validated assessment instruments for the study population, which have demonstrated adequate psychometric properties in previous studies. Prior authorization was requested and obtained from the respective authors for the use of the Portuguese versions of these instruments.

- The Mental Health Literacy Questionnaire—Short Version (MHLq-SVa) [15,31] is a brief instrument designed to evaluate an individual’s MHL. It assesses four domains: (1) knowledge of mental health problems (items 7, 10–12, 14–15); (2) erroneous beliefs/ stereotypes (reverse-coded items 2, 5, 6); (3) help-seeking and first aid skills (items 4, 8, 16); and (4) self-help strategies (item 1, 3, 9, 13). Participants were asked to evaluate each item by selecting the option that best reflected their level of agreement, using a 5-point scale (1 = Strongly disagree, 2 = Disagree, 3 = Neither agree nor disagree, 4 = Agree, and 5 = Strongly agree). The total score results from the sum of the scores obtained on the 16 items of the scale. Higher scores indicate greater MHL, reflecting better understanding, more positive attitudes, and more effective help-seeking behaviors. The MHLq-SVa is a reduced version of a previously validated questionnaire for assessing MHL, based on a confirmatory factor analysis (CFA) tested in a validation study [30] conducted with samples of senior school and undergraduate students from six countries, including Portugal. The correlations between the domains and the total (−0.29 to 0.92) corroborated the internal consistency, as did the Cronbach’s α values (total = 0.82; domains = 0.66 to 0.72). In the current study, Cronbach’s α values were similar for the total (α = 0.83) and the domains (range: 0.60 to 0.75).
- Academic Life Satisfaction (ALS) [32] is a self-report instrument designed to assess students’ overall satisfaction with their academic experience. Based on a CFA, this scale integrates two dimensions: (1) personal satisfaction: internal—students’ skills and abilities, perception of academic performance, and relationships with peers and professors (items 5–8); and (2) satisfaction with the academic environment: external—physical and pedagogical environment, interest and commitment to the course, the institution’s extracurricular activities, study conditions (items 1–4). Responses are provided on a 5-point Likert scale (1 = strongly disagree to 5 = strongly agree). Total scores are the sum of the eight item values (range: 8–40), with higher scores indicating greater perceived academic satisfaction. In the validation study with a sample of Portuguese university students, the ALS showed good content and criterion validity, as well as adequate reliability (total α = 0.80; dimensions: α > 0.70), consistent with the values found in the present study.
- The Subjective Happiness Scale (SHS) [33,34] is a brief, unidimensional self-report measure designed to assess an individual’s overall subjective happiness by considering how happy a person feels and how they compare their happiness to others. It comprises four items, and respondents are asked to indicate the extent to which each statement characterizes them, using a seven-point visual analogue scale anchored by two opposing statements reflecting the presence or absence of happiness. A single composite score for global subjective happiness is computed by averaging responses to the four items, yielding a range from 1.0 to 7.0, with higher scores reflecting greater happiness. In the validation study of the Portuguese version [33], conducted with a convenience sample of young people and adults, the CFA supported a single-factor structure, and convergent and discriminant validity, as well as internal consistency (α = 0.76), as well as presented appropriate values, comparable to those of the original study, although the internal consistency in the present study was slightly lower (α = 0.63).
- The Psychological Well-Being Scale (PWBS) [25,35] is a self-report instrument designed to assess multiple dimensions of an individual’s PWB. The scale includes 18 items with a 5-option Likert-type scale and evaluates six domains: autonomy (items 1, 7 and 13); environmental mastery (items 2, 8 and 14); personal growth (items 3, 9 and 15); positive relationships with others (items 4, 10 and 16); purpose in life (items 5, 11 and 17); and self-acceptance (items 6, 12 and 18). Responses are rated on a 6-point Likert scale (1 = strongly disagree to 6 = strongly agree), with 10 items reverse-scored. Domain scores range from 3 to 18, and the total score is obtained by summing all items (range: 18–108), with higher scores reflecting higher levels of PWB. The Portuguese version was validated in a heterogeneous sample of young and older adults, and its six-factor structure of Carol Ryff’s theoretical model was corroborated, with evidence of construct, convergent, and discriminant validity, as well as adequate internal consistency (total α = 0.82, domains α = 0.70–0.85). In the current study, the total α was slightly higher (α = 0.87), although the domain α values were somewhat lower (α = 0.42–0.69).
- The Depression Anxiety Stress Scale—21 (DASS-21) [36,37] was developed as a self-report questionnaire to assess symptoms related to depression, anxiety, and stress. The scale contains 21 items, with seven items for each dimension. Participants rated how well each statement applied to them in the past week, using a 4-point Likert scale, in relation to the severity and frequency of symptoms in the past 7 days (0—“did not apply to me at all”, 3—“applied to me most of the time”). The total score resulted from the sum of the scale items, ranging from 0 to 63 points and from 0 to 21 in each domain. Higher scores indicate higher levels of symptoms of depression, anxiety, or stress. Pais-Ribeiro et al. [37] carried out the Portuguese adaptation of the reduced version of this scale in a convenience sample of Portuguese young adults (ages: 18–95 years), with the factor analysis confirming the structure of the three factors and an adequate internal consistency, with a total α = 0.87, although the α per subscale was slightly lower (depression α = 0.85, anxiety α = 0.74, stress α = 0.81). In this study, the alpha values were significantly higher for the total (α = 0.95) and for the subscales (α = 0.88–0.89).

### 2.4. Ethical Statement

The current study was approved, received ethical approval from the Research Ethics Committee of the higher-education institution where it was conducted (approval number: CE/IPSN/CESPU-48/24), and was carried out in accordance with the Declaration of Helsinki.

At the time of enrolment, all potential participants received detailed information about this research, namely about its objectives, methodological procedures, strategies to guarantee the confidentiality and anonymity of the data, the voluntary nature of participation, the absence of compensation or incentive, and potential benefits and risks of participation, with indication of the contact of the institution’s psychological support service and/or the project coordinator in case of discomfort during questionnaire completion and/or questions about the project. This information was available in the questionnaire hosted on the institution’s own online platform. Informed consent was obtained electronically in this same online form. At the end, a copy of the consent form was available to the participants.

Data confidentiality was ensured through an anonymization process that prevented the identification of participants. All data entered on the platform was automatically exported to a database built for this project, ensuring confidentiality and anonymity (e.g., no ID or email identification). Only the project coordinator and the team member responsible for data processing have access to this database.

Regarding the storage of this data, it will be deleted following the publication of all primary results arising from this project.

### 2.5. Data Processing and Analysis

Statistical analysis was run using IBM SPSS Statistics for Windows, version 30.0 (IBM Corp., Armonk, NY, USA). Initially, a descriptive analysis of the variables was performed according to their nature. For qualitative variables, absolute (N) and relative (%) frequencies were reported. For quantitative variables, the mean (M), standard deviation (SD), minimum and maximum values were presented, or, in the case of skewed distributions with outliers, the median (Mdn) and interquartile range (IQR) were reported.

Data was screened for missing values prior to the statistical analysis. Missing data was minimal (less than 5% across variables) and was handled using listwise deletion (complete-case analysis), whereby participants with missing values on the variables of interest were excluded from the analyses, which is generally considered acceptable when the proportion of missing observations is small [38].

The internal consistency of the standardized instruments was assessed using Cronbach’s alpha coefficient (α), with values above 0.70 suggesting good internal consistency, while >0.90 indicates very high internal consistency and excellent reliability [39,40].

A multivariable linear regression analysis was performed to examine the independent associations between the explanatory variables and MHL. A backward elimination procedure was applied to obtain a parsimonious model. However, it should be noted that variable selection based on *p*-value thresholds and backward elimination procedures may increase the risk of overfitting and model instability, and therefore the results should be interpreted with caution [41,42]. Prior to interpreting the regression model, the assumptions of linear regression were evaluated. Linearity and homoscedasticity were assessed through inspection of scatterplots and residual plots, normality of residuals was examined using Q–Q plots, and multicollinearity was assessed using variance inflation factors (VIFs). Influential observations were evaluated using standardized residuals, Cook’s distance and influence statistics. After excluding cases with missing values for the variables included in the model, the regression analysis included a total of 276 participants. This sample size exceeds commonly recommended thresholds for multiple regression models, suggesting adequate statistical power to detect moderate associations according to established guidelines [43]. MHL (MHLq-SVa) was defined as the dependent variable, while sociodemographic characteristics, academic status, and health status were entered as independent variables. The selection of independent variables was based on previous bivariate analyses (Mann–Whitney and Kruskal–Wallis tests), considering *p* < 0.10 as the inclusion criterion in the model. For the nonparametric tests, effect sizes were also calculated: r for the Mann–Whitney test (|r| ≈ 0.10 = small; |r| ≈ 0.30 = moderate; |r| ≥ 0.50 = large) and η^2^ for the Kruskal–Wallis test (|η^2^| ≈ 0.01 = small; |η^2^| ≈ 0.06 = moderate; |η^2^| ≥ 0.14 = large), following Cohen’s (1988) guidelines [43]. Given the number of bivariate comparisons performed, these analyses were considered exploratory and the results were interpreted with caution to minimize the risk of Type I error. A significance level of 5% was assumed for all statistical tests.

## 3. Results

### 3.1. Characteristics of Study Participants

This study includes a sample of 306 undergraduate health students, with an average age of 21.6 (SD = 4.88), most of whom were female (79.4%) and single (95.1%). More than half (58.8%) were nursing students, with 42.2% attending the 4th year. In almost 90% of cases, this course was their first choice, and a small fraction (8.8%) already had a previous degree (Table 1).

Regarding lifelong support needs for MH problems, 47.7% received support from a health service or professional at some point in their lives, and about 13.4% were currently receiving support. During the MH support period, about 33% needed psychotropic medication. Almost 13% had been diagnosed with a mental illness or disorder, and only 4.6% needed to be hospitalized due to mental problems. Furthermore, 8.5% of participants were taking psychotropic medication for anxiety, 6.9% for sleep problems, and 5.9% for depression. About 37% had a family history of mental disorders (Table 2).

### 3.2. Multidimensional Evaluation of Study Participants

The multidimensional assessment of the study participants revealed generally high levels of MHL and relatively positive psychosocial indicators. The median total score of the MHLq-SVa was high (Mdn = 70; range: 30–80), with the domain knowledge of mental health problems presenting the highest median values among the subscales. Regarding PWB, the highest scores were observed in the personal growth, purpose in life, and self-acceptance domains. Participants also reported relatively high levels of subjective happiness and academic life satisfaction, while symptoms of stress were the most prominent dimension within the DASS-21 scale. Descriptive statistics and internal consistency coefficients for the instruments used are presented in Table 3.

As can be observed in Table 3, the internal consistency evaluated with Cronbach’s alpha coefficient was moderate to high for most of the totals and domains of the assessment instruments included in research protocol. Although most instruments showed acceptable internal consistency, some subscales presented relatively modest Cronbach’s alpha values. This may partly be explained by the limited number of items included in certain subscales, as Cronbach’s alpha is known to be sensitive to scale length. Therefore, the results involving these dimensions should be interpreted with caution.

### 3.3. Factors Associated with MHL (Bivariate Analysis)

To identify factors associated with MHL among undergraduate health students, an analysis was conducted to examine the relationship between MHL (MHLq-SVa) and the sociodemographic characteristics, academic status, mental health history, and psychological factors of the participants in the sample, which include psychological well-being and happiness. The findings of this analysis are presented in detail in Tables S1–S3 in the Supplementary Materials.

In relation to sociodemographic characteristics, only a marginally significant difference in MHL levels based on sex was found, with female participants scoring higher on the MHLq-SVa instrument (Mdn = 69 vs. 71; *p* = 0.063) than male participants (Table S1).

Regarding the academic situation, it was found that students who already had a degree had higher levels of MHL than those without a previous degree (Mdn = 74 vs. 70; *p* = 0.003). In addition, statistically significant differences were found regarding place of residence during the school term, with students living at home showing higher levels of MHL (Mdn = 71 vs. 68; *p* = 0.046). Statistically significant differences were also observed between students for whom the course was their first choice and those for whom it was not, with the former group showing higher levels of MHL (Mdn = 71 vs. 68; *p* = 0.046). No differences were found for the other variables related to academic status (Table S2).

Regarding the analysis of the relationship between MHL and MH history, it was found that students who required psychotropic medication during the period in which they received support for psychological and MH problems demonstrated higher levels of MHL compared with those who did not require pharmacological intervention (Mdn = 72.5 vs. 70; *p* = 0.029). Moreover, students who report currently taking medications for anxiety, depression, and sleep problems presented higher levels of MHL (Mdn = 71 vs. 70; *p* = 0.037) (Table S3).

However, it should be noted that given the number of bivariate comparisons performed, these analyses were considered exploratory and the results were interpreted with caution to minimize the risk of Type I error.

With regard to the standardized assessment instruments included in the data collection protocol, only the 271 participants with responses common to all instruments (listwise exclusion of cases) were considered for analysis of the correlation between MHLq-SVa total score, (MHL measure), and the total of the other instruments: PWBS (psychological well-being), SHS (happiness), ALS (satisfaction with academic life), and DASS (depressive/anxious symptoms).

The results indicate that MHL was positively associated with PWBS (r_s_ = 0.280; *p* < 0.01), indicating a small-to-moderate effect size. A statistically significant but weak positive association was observed with SHS (r_s_ = 0.164; *p* < 0.01), corresponding to approximately 2.7% of shared variance, suggesting limited practical significance. No statistically significant association was found between MHL and ALS (rs = 0.115, *p* > 0.05), nor with depressive, anxiety, and stress symptoms (rs = −0.005, *p* > 0.05). Overall, although some associations reached statistical significance, the magnitude of the correlations indicates that the shared variance between MHL and these variables is generally small [43].

From further analysis, it was found that the total of PWBS correlated negatively with depressive and/or anxiety symptoms (r_s_ = −0.492; *p* < 0.01), indicating a strong inverse relationship. A moderate positive correlation was also observed between PWBS and academic satisfaction (r_s_ =0.409; *p* < 0.01) and subjective happiness (r_s_ = 0.359; *p* < 0.01). The remaining correlations were weak in magnitude, although some were statistically significant (Table 4).

### 3.4. Multivariable Analysis of Factors Associated with MHL

The following variables showed statistically significant associations (*p* < 0.10) with MHL in the prior bivariate analysis and were therefore included in the corresponding blocks of the model: sociodemographic (gender, *p* = 0.063); academic status (residence during the academic year, *p* = 0.045; current course was first choice, *p* = 0.046; having a previous degree, *p* = 0.003); health-related variables (take psychotropic medication, when followed by a healthcare professional due MH problems, *p* = 0.029; current use of medication for sleep/anxiety/depression, *p* = 0.037; having a family member with a mental illness/disorder, *p* = 0.081); and variables related to psychological assessment constructs (PWBS, *p* < 0.01; subjective happiness, *p* < 0.01).

Regarding the verification of the model assumptions, the normality of the unstandardized residuals was confirmed by the Kolmogorov–Smirnov test with Lilliefors correction, which indicated no significant deviations from normality (D = 0.041; *p* = 0.302). Homoscedasticity was assessed using the scatter plot of standardized residuals against standardized predicted values, showing a random distribution of points around the zero line. The independence of the residuals was supported by the Durbin–Watson statistic (d = 1.81). No evidence of multicollinearity was detected, with all VIF values below 1.3. Diagnostic statistics indicated no major violations of regression assumptions. Standardized residuals were within acceptable limits (−2.18 to 2.24), Cook’s distance values were low (maximum = 0.047), and leverage values did not indicate the presence of influential observations. Five observations with unusually large studentized residuals (|studentized residual| > 3) were excluded from the regression analysis to avoid undue influence on the model estimates. Although this approach is commonly used to reduce the influence of extreme observations, no formal sensitivity analysis was conducted to assess the impact of these exclusions, and results should therefore be interpreted with caution.

The final linear regression model explained approximately 17.5% of the variance in MHL levels (R^2^ = 0.175; adjusted R^2^ = 0.160), suggesting that the identified variables are associated with MHL, although these associations are modest in magnitude. According to this model, students who indicated that the course corresponded to their first choice had significantly higher levels of MHL (B = 2.954; β = 0.167; *p* = 0.003), as did those who stated that they already had a degree (B = 4.626; β = 0.219; *p* < 0.001). In addition, current use of medication for sleep, anxiety and/or depression was positively associated with MHL (B = 2.115; β = 0.173; *p* = 0.003). PWB, measured by the total PWBS (higher scores on this measure correspond to higher levels of PWB), was also positively associated with MHL (B = 0.115; β = 0.269; *p* < 0.001), which was the strongest associated factor in the model (β = 0.269) (Table 5).

## 4. Discussion

The description of MHL levels among undergraduate health students was the main objective of this study and, according to the results of the MHLq-Sva, the median total score was 70, with the domain “Knowledge about mental health problems” having the highest median value. Considering that total score on the MHLq-Sva ranges from 30 to 80 and that higher scores correspond to higher levels of MHL, it can be inferred that this sample reveals a high level of MHL.

These results, including the dimension of MHL that stood out, can be explained in part by the sample of students enrolled in health courses, mostly nursing, and attending the final years of the course; so, it is expected that content on health and mental illnesses/disorders will be addressed in the syllabus. These findings may also reflect whether high MHL scores represent genuine competence or are influenced by socially desirable responding, particularly among health student populations. However, studies on MHL in undergraduate students (some with nursing students) and conducted in different countries point to a trend of divergent results, ranging from moderate [44,45] to high levels of MHL [46,47]. This can be attributed to the heterogeneity of the assessment instruments used but also to differences between countries in terms of levels of development, education, and quality of mental health services [44].

Another objective of this study was to identify factors related to academic life, MH and psychological factors that may be associated with MHL. In prior bivariate analysis, female gender, living at home during the academic year, having a previous degree, taking psychotropic medication, being followed by a healthcare professional due to mental health problems, and higher levels of subjective happiness were statistically positively associated with MHL. However, in the final linear regression model, it was found that students who indicated that the course matched their first choice had significantly higher levels of MHL. Furthermore, current use of medication for sleep, anxiety, and/or depression was positively associated with MHL. But it is PWB that emerged as the strongest associated factor in the model, with high levels in this domain being associated with higher levels of MHL.

It is noteworthy that the final regression model explained 17.5% of the variance in the MHL (R^2^ = 0.175), indicating that a substantial proportion of the variability remains unexplained, which is not uncommon in behavioral and psychosocial research, where complex outcomes are influenced by multiple interacting determinants that may not have been fully assessed or integrated into the cross-sectional model used [43].

Analyzing these results in more detail, it is worth noting the predominance of female participants and their young age, characteristics commonly reported in health-related degree programs such as nursing, where women are typically overrepresented. This demographic profile is relevant since gender and age are important determinants of MHL and help-seeking behavior. Studies have shown that women generally present higher levels of MHL and are more likely to recognize symptoms and seek professional help than men, who tend to report greater stigma and lower self-efficacy toward help-seeking [27,48]. However, despite this overrepresentation, gender was not significantly associated with MHL in the regression analysis. This could be due to the relatively homogenous sample in terms of age and educational background, which may have reduced variability in MHL.

Regarding academic life, most participants reported that their current program was their first choice, and a small subgroup had previous academic experience, reflecting vocational maturity. However, in the regression analysis, only first-choice status was significantly associated with MHL, which may reflect that it is more influential than other academic or demographic factors assessed. It may also reflect that students with greater academic engagement and clearer career goals may exhibit better emotional regulation and self-awareness, factors associated with better coping, well-being, and higher levels of MHL [1,21].

As concerns MH history, almost half of the sample had received or were receiving psychological support, and 13.4% were undergoing treatment at the time of data collection. These values are slightly higher than those commonly reported in international literature, where estimates range between 30% and 45% [49,50]. In the linear regression model, it was verified that students with current use of medication for sleep, anxiety, and/or depression revealed higher levels of MHL. This finding may reflect a higher awareness of MH among health students, who are more frequently exposed to self-care, empathy, and emotional regulation topics [51]. In addition, students with a history of MH problems or support needs for such problems may be more aware of MH issues, as well as being better able to recognize symptoms and seek help earlier, which may explain their higher levels of MHL [52,53].

Furthermore, approximately one-third of the sample reported a family history of mental illness, which, according to Martínez Líbano et al. [27], is associated with greater recognition of MH problems and more positive attitudes toward help-seeking. Such findings highlight that MHL encompasses not only knowledge but also attitudes, lived experiences, and behavioral readiness to seek assistance, influenced by factors such as stigma and emotional intelligence [54].

As already mentioned, PWB was the most robust factor associated with MHL. According to Pan et al. [21], well-being has emerged as an indicator linked to MH, receiving increasing attention from researchers in recent years, with some studies reporting a correlation between these two concepts. For example, a cross-sectional study [55] with 1628 Pakistani undergraduate students from various public universities found a positive correlation between PWB and MHL, suggesting that MHL plays a significant role in mediating the relationship between MH status and well-being. More recently, Usenik and Kranjec [56] also reported that MHL was positively associated with well-being and negatively associated with anxiety and depression symptoms in a sample of 219 Slovenian dual-career athletes (which includes adolescents and university students). According to the results of students from different higher-education institutions, the authors concluded that students with lower levels of MHL were more psychologically vulnerable, while higher literacy was associated with positive MH (indicators include positive emotions, healthy relationships, and personal functioning) [30]. Another national study [57] reported those undergraduate older students, with healthy behaviors, low psychological vulnerability, and positive mental well-being had higher MHL, whereas psychological vulnerability and poor health habits were linked to lower literacy.

Nevertheless, some studies did not find a direct association between MHL and PWB, but rather with help-seeking behavior [16,46,47]. In this view, the authors explain that this behavior may be associated with improved well-being and lower psychological distress among students, potentially mediating the association between MHL and these outcomes [46,47]. On the other hand, the absence of a relationship between MHL and PWB in some studies may be explained by the potential influence of confounding variables, such as personality traits, academic performance, and socioeconomic level, which may not always have been controlled for.

Overall, the association between MHL and PWB appears to be correlational rather than causal. Greater knowledge about MH, including knowing how to identify the early signs, when professional support is needed and how and where to seek help, as well as having fewer stigmatizing attitudes towards MH, can help these students better cope with stressful situations and MH problems, ultimately enhancing their PWB and happiness [1]. Similarly, students with higher PWB may have more resources or motivation to engage with mental health information. This suggests a potential bidirectional or mutually reinforcing relationship, although the underlying mechanisms remain unclear.

Additional findings from this study emerged from the multidimensional assessment conducted and should be addressed, namely the high prevalence of anxiety, stress, and depressive symptoms among students, which is consistent with findings from recent Portuguese and international research. Amaro et al. [10] found that over 60.2% of Portuguese undergraduate students experienced moderate-to-severe anxiety, while 61% showed depressive symptoms. Similarly, Vidović et al. [58] and Ahmed et al. [59] reported alarming rates of psychological distress among European and global university populations. The identified age group of the study sample corresponds to a developmental stage characterized by multiple academic, social, and personal transitions, which may contribute to increased vulnerability to stress and emotional disorders [10,59]. In addition, these findings emphasize the importance of promoting preventive and literacy-based strategies in higher education.

This study has some strengths that should be highlighted. Firstly, this study contributes to the further investigation of MHL among undergraduate health students. In addition, it employs a comprehensive analytic strategy that goes beyond descriptive statistics, using multivariate analyses to explore the relationships between MHL and socio-demographic characteristics, mental health history, academic situation, and psychosocial dimensions, highlighting variables that have been little explored, such as PWB and happiness, which may provide a more nuanced conceptual understanding of students’ mental health and the factors that influence MHL.

Furthermore, the findings underscore the practical relevance of this study, highlighting the need for higher-education institutions to implement structured, evidence-based programs to promote MHL through psychoeducational interventions or other initiatives in partnership with MH services.

Despite the relevance of these findings, some limitations must be acknowledged. Most notably, the use of convenience sampling within a single institution, with a predominance of female and nursing students, may limit the external validity and introduce potential self-selection bias. The cross-sectional design limits generalization to larger or more diverse populations. Additionally, reliance on self-report measures increases the risk of social desirability and recall bias, while participants’ prior interest in mental health may have contributed to inflated MHL levels. The relatively high MHL scores also suggest a possible ceiling effect, reducing variability and potentially weakening observed associations. Finally, the modestly explained variance in the regression model indicates that other unmeasured variables may also influence MHL. In addition, the use of complete-case analysis (no comparison was made between included and excluded participants) and multiple bivariate tests use may have introduced selection bias and increased the risk of Type I error, and the lack of analysis of interaction effects limits a deeper understanding of the relationships between variables. Additionally, the exclusion of outliers without formal sensitivity analysis may have influenced the stability of the regression estimates.

Nevertheless, while these results align with international evidence [21,51], they should be interpreted considering this study’s limitations. Despite these constraints, the findings underscore the importance of considering MHL as a strategic axis of highe- education MH policies, combining educational, psychosocial, and institutional approaches in accordance with modifiable associated factors.

## 5. Future Implications

Higher-education institutions may consider developing and implementing structured, evidence-based programs to promote MHL through psychoeducational interventions, peer-led initiatives, and the integration of MHL into existing academic and student support structures. For example, incorporating brief MHL components into existing courses or mentoring activities, as well as strengthening referral channels between university and mental health services, may represent viable and context-appropriate approaches. Given the characteristics of the present sample, these strategies may be particularly relevant for the early identification of students experiencing psychological distress and for normalizing help-seeking in the university context. These initiatives may also effectively reduce stigma, increase demand for help, and develop adaptive coping skills.

Future research should prioritize longitudinal designs to examine how MHL and psychological well-being co-vary over time, while intervention studies could analyze the impact of targeted MHL components embedded within academic settings. It will also be important to explore mediating and moderating variables, including self-stigma, emotional intelligence, resilience, and perceived social support, to better understand the mechanisms underlying MHL interventions. Comparative studies across academic fields could further elucidate how professional identity and academic culture influence MHL and attitudes toward psychological support. Additionally, replication across more diverse and balanced samples would help to clarify the generalizability of these findings.

In the long term, integrating MHL within institutional policies and academic curricula may foster a culture of empathy, awareness, social connection, and shared responsibility for MH across university communities.

## 6. Conclusions

The findings of this study suggest that PWB is modestly associated with MHL among undergraduate health students, contributing to the understanding of how these constructs are related within undergraduate health students. Other factors related to academic situation and mental health history also revealed an association with MHL. All these results support the conceptualization of MHL as a multidimensional construct that encompasses knowledge, attitudes, and behaviors, which may influence aspects such as symptom recognition, stigma reduction, and help-seeking intentions. However, the relatively low explained variance highlights that other unexamined factors are likely to play a significant role in these outcomes. Moreover, the sample profile, predominantly female, young, and with a substantial proportion of students who had previously sought psychological support, reflects both heightened emotional vulnerability and increased MH awareness; so, these characteristics should be interpreted with caution due to possible sampling biases.

The high prevalence of anxiety and depressive symptoms observed in this population underscores the relevance of promoting mental health within university settings. Rather than implying causal relationships, these findings point to the potential value of integrating MHL into institutional strategies, with emphasis not only on symptom identification but also on strengthening emotional regulation, self-efficacy, and social connectedness as part of a boarder approach to supporting student psychological resilience and well-being.

## Figures and Tables

**Table 1:** Sociodemographic data and academic situation characterization of the sample (n = 306).

	N	%
Age (years), Mean = 21.6 (SD = 4.88)		
**Sex**		
Male	63	20.6
Female	243	79.4
**Marital status**		
Single	291	95.1
Married	11	3.6
Divorced	4	1.3
**Degree studies**		
Nursing	180	58.8
Physiotherapy	112	36.6
Other	14	4.6
**Year of study**		
1st year	63	20.6
2nd year	62	20.3
3rd year	129	42.2
4th year	52	17.0
**Course was the first option**		
No	32	10.5
Yes	274	89.5
**Prior degree**		
No	279	91.2
Yes	27	8.8

SD = standard deviation.

**Table 2:** Mental health history characterization of the sample (n = 306).

	N	%
**Lifelong MH support**		
Never	155	50.7
Yes, but I’m done	98	32.0
Yes, I’m still going	48	15.7
I’d rather not answer	5	1.6
**Taking psychotropic medication during the MH support period**		
No	232	75.8
Yes, I still take this medication	30	9.8
Yes, but I no longer take this medication	40	13.1
I’d rather not answer	4	1.3
**Diagnosis of mental illness or disorder**		
No	255	83.3
Yes	39	12.7
I’d rather not answer	12	3.9
**Hospitalization due to mental illness/disorder**		
No	291	95.1
Yes	14	4.6
I’d rather not answer	1	0.3
Current MH support		
No	262	85.6
Yes	41	13.4
I’d rather not answer	3	1.0
**Currently taking medication for sleep problems**		
No	256	83.7
Yes	21	6.9
Only during exam period	26	8.5
I prefer not to answer	3	1.0
**Currently taking medication for anxiety**		
No	245	80.1
Yes	26	8.5
Only during exam period	33	10.8
I prefer not to answer	2	0.7
**Currently taking medication for depression**		
No	283	92.5
Yes	18	5.9
Only during exam period	3	1.0
I prefer not to answer	2	0.7
**Nuclear family member with a mental illness or disorder**		
No	190	62.1
Yes, but you don’t live with that relative	57	18.6
Yes, and I live with that relative	57	18.6
I prefer not to answer	2	0.7

MH = mental health.

**Table 3:** Descriptive statistics and internal consistency of measurement instruments.

	Mdn	Min.	Max.	Nº Items	Cronbach’s Alpha (α)
**MHLq-Sva** ^(n = 305)^					
Knowledge of Mental Health Problems	25.0	11	30	6	0.75
Erroneous Beliefs/Stereotypes	14.0	3	15	3	0.60
First Aid and Help-Seeking Skills	13.0	5	15	3	0.73
Self-Help Strategies	18.0	5	20	4	0.68
Total	70.0	30	80	16	0.83
**PWBS** ^(n = 284)^					
Autonomy	12.0	3	18	3	0.56
Environmental Mastery	11.0	4	18	3	0.59
Personal Growth	15.0	6	18	3	0.56
Positive Relations with Others	12.0	4	18	3	0.42
Purpose in Life	14.0	3	18	3	0.69
Self-acceptance	14.0	6	18	3	0.64
Total	77.0	39	106	18	0.87
**SHS** ^(n = 281)^					
Total	4.5	1	7	4	0.63
**ALS** ^(n = 279)^					
Satisfaction with the Academic Environment	14.0	6	20	4	0.72
Personal Satisfaction	16.0	5	20	4	0.77
Total	30.0	11	40	8	0.83
**DASS** ^(n = 271)^					
Depression	4.0	0	21	7	0.89
Anxiety	4.0	0	21	7	0.88
Stress	7.0	0	21	7	0.89
Total	14.0	0	63	21	0.95

MHLq-SVa—Mental Health Literacy Questionnaire; ALS—Academic Life Satisfaction; SHS—Subjective Happiness Scale; PWBS—Psychological Well-Being Scale; DASS—Depression Anxiety Stress Scale; Mdn—Median; Min.—Minimum; Max.—Maximum.

**Table 4:** Spearman’s correlation matrix between MHL and PWB, happiness, satisfaction with academic life, and depressive/anxious symptoms (N = 271).

	MHLq-SVa	PWBS	SHS	ALS	DASS
PWBS	0.280 **	1			
SHS	0.164 **	0.359 **	1		
ALS	0.115	0.409 **	0.235 **	1	
DASS	−0.005	−0.492 **	−0.198 **	−0.228 **	1

MHLq-SVa—Mental Health Literacy Questionnaire; ALS—Academic Life Satisfaction; SHS—Subjective Happiness Scale; PWBS—Psychological Well-Being Scale; DASS—Depression Anxiety Stress Scale; Spearman’s correlation coefficient (** *p* < 0.01).

**Table 5:** Coefficients of the final multiple linear regression model for Mental Health Literacy (N = 276).

	B	Standard Error B	β	T
Course was the first option	2.954	0.996	0.167	2.966
Prior degree	4.626	1.186	0.219	3.899
Psychotropic medication	2.115	0.703	0.173	3.008
Psychological Well-Being (PWBS Total)	0.115	0.025	0.269	4.658

Dependent variable = Mental Health Literacy (MHLq-SVa). R^2^ = 0.175; R^2^ Adjusted = 0.160. PWBS—Psychological Well-Being Scale.

## Data Availability

The data presented in this study are available on request from the corresponding author. The data are not publicly available due to privacy and ethical restrictions.

## Supplementary Materials

The following supporting information can be downloaded at: https://www.mdpi.com/article/10.3390/nursrep16040109/s1, Table S1: Comparison of Mental Health Literacy according to Sociodemographic Characteristics (N = 305); Table S2: Comparison of Mental Health Literacy according to Students’ Academic Status (N = 305); Table S3: Comparison of Mental Health Literacy, according to Students’ Mental Health History (N = 305).

## Public Involvement Statement

There was no public involvement in any aspect of this research.

## Guidelines and Standards Statement

This manuscript was prepared generally based on the STROBE (Strengthening the Reporting of Observational Studies in Epidemiology) guidelines for observational studies with a cross-sectional design.

## Abbreviations

The following abbreviations are used in this manuscript:

ALS	Academic Life Satisfaction
CFA	Confirmatory factor analysis
COVID-19	Coronavirus disease 2019
DASS-21	Depression Anxiety Stress Scale-21
IQR	Interquartile range
M	Mean
Max.	Maximum
Mdn	Median
Min.	Minimum
MH	Mental health
MHL	Mental health literacy
MHLq-SVa	MHLq-SVa—Mental Health Literacy Questionnaire
PWB	Psychological well-being
PWBS	Psychological Well-Being Scale
SD	Standard deviation
SHS	Subjective Happiness Scale
VIFs	Variance inflation factors
α	Cronbach’s alpha coefficient
