# Supplementary material for: Factors Associated with Mental Health Literacy Among Undergraduate Health Students in Portuguese Higher Education: The Role of Psychological Well-Being
Source: Nurs Rep. 2026 Mar 27;16(4):109. doi: 10.3390/nursrep16040109 (PMC13118875; doi:10.3390/nursrep16040109)
Supplement: Supplementary file 1 [file nursrep-16-00109-s001.zip › nursrep-4156349-supplementary.pdf]

Supplementary Materials

Table S1. Comparison of Mental Health Literacy according to Sociodemographic Characteristics (N=305)

	Mental Health Literacy (MHLq-Sva)					
	Mean Rank	Mdn (IQR)	Test statistic	Z	p-value	r
Sex						
Male (n=63)	134.60	69.0 (8.0)	U=6463.50	-1.86	0.063	0.11
Female (n=242)	157.79	71.0 (9.0)				
Age (age group)						
≤20 years (n=151)	152.68	70.0 (8.0)	U=11578.00	-0.06	0.949	0.004
>20 years (n=154)	153.32	71.0 (9.0)				
Marital Status						
Other (n=15)	185.47	73.0 (8.0)	U=1688.00	-1.46	0.143	0.08
Single (n=290)	151.32	70.0 (8.0)				
Who do you currently live with?				df	p-value	$\eta 2$
With parents (n=96)	155.20	70.0 (9.0)	H=1.98	2	0.372	0.00
With parents and siblings (n=121)	159.13	71.0 (8.0)				
Others (n=88)	142.16	70.0 (9.0)				

MHLq-SVa—Mental Health Literacy Questionnaire; Mdn - Median; IQR - Interquartile range (P75-P25); U - Mann-Whitney test statistic; Z - Standardized Mann-Whitney test statistic; r - Effect size; H - Kruskal-Wallis's test statistic; df - Degrees of freedom; $\eta 2$ - Effect size.

Table S2. Comparison of Mental Health Literacy according to Students' Academic Status (N=305)

	Mental Health Literacy (MHLq-Sva)					
	Mean Rank	Mdn (IQR)	Test statistic	Z	p-value	r
Degree studies				df		$\eta 2$
Nursing (n=180)	153.29	70.0 (9.0)	H=0.02	2	0.992	0.00
Physiotherapy (n=112)	152.31	70.5 (9.0)				
Other courses (n=13)	154.96	71.0 (10.0)				
Year of the course you are attending				df		$\eta 2$
1st year (n=63)	157.25	71.0 (7.0)	H=1.37	3	0.713	0.00
2nd year (n=61)	160.44	70.0 (10.0)				
3rd year (n=129)	151.69	71.0 (8.0)				
4th year (n=52)	142.37	69.5 (11.0)				
Student status						
Worker student/High competition athlete (n=54)	163.66	71.0 (8.0)	U=6201.50	-0.98	0.327	0.06
Student (n=251)	150.71	70.0 (8.0)				
During the academic term, you reside:						
Outside the home (n=47)	129.27	70.0 (9.0)	U=4947.50	-2.01	**0.045**	0.12
At home (n=258)	157.32	71.0 (9.0)				
Did this course correspond to your first option?						
No (n=31)	123.15	68.0 (6.0)	U=3321.50	-1.99	**0.046**	0.11
Yes (n=274)	156.38	71.0 (9.0)				
Do you have any prior degree?						
No (n=279)	148.39	70.0 (8.0)	U=2341.00	-2.99	**0.003**	0.17
Yes (n=26)	202.46	75.0 (9.0)				
Are you currently doing an internship?						
No (n=298)	153.44	70.0 (9,0)	U=912.50	-0.57	0.571	0.03
Yes (n=7)	134.36	71.0 (16,0)				

MHLq-SVa—Mental Health Literacy Questionnaire; Mdn - Median; IQR - Interquartile range (P75-P25); U - Mann-Whitney test statistic; Z - Standardized Mann-Whitney test statistic; r - Effect size; H - Kruskal-Wallis's test statistic; df - Degrees of freedom; $\eta 2$ - Effect size.

Table S3. Comparison of Mental Health Literacy, according to Students' Mental Health History (N=305)

	Mental Health Literacy (MHLq-Sva)					
	Mean Rank	Mdn (IQR)	Test statistic	Z	p-value	r
Throughout your life, have you needed to seek help from a health professional or service for psychological or psychiatric problem or illness?						
No (n=155)	143.85	70.0 (8.0)	U=10207.50	-1.37	0.170	0.08
Yes (n=145)	157.60	71.0 (9.0)				
During this follow-up period, have you taken any type of psychotropic medication (antidepressants, anxiolytics, benzodiazepines, or antipsychotics)?						
No (n=231)	144.99	70.0 (8.0)	U=6697.00	-2.18	**0.029**	0.13
Yes (n=70)	170.83	72.5 (9.0)				
Have you ever been diagnosed with a psychiatric illness or disorder?						
No (n=254)	149.77	71.0 (9.0)	U=4249.00	-1.43	0.152	0.08
Yes (n=39)	128.95	68.0 (9.0)				
Throughout your life, have you needed to be hospitalized for a psychological or psychiatric problem or illness?						
No (n=290)	151.50	70.0 (9.0)	U=1740.50	-0.90	0.367	0.05
Yes (n=14)	173.18	71.5 (9.0)				
Are you currently being treated by a health professional for a psychological or psychiatric problem or illness?						
No (n=261)	150.53	70.0 (9.0)	U=5097.00	-0.49	0.625	0.03
Yes (n=41)	157.68	71.0 (8.0)				
Taking any medication for sleep/anxiety/depression						
No (n=223)	146.62	70.0 (9.0)	U=7719.50	-2.09	**0.037**	0.12
Yes (n=82)	170.36	72.5 (8.0)				
Do you have any member of your nuclear family with a psychological/psychiatric illness?						
No (n=158)	144.52	70.0 (8.0)	U=10273.00	-1.74	0.081	0.10
Yes (n=147)	162.12	71.0 (9.0)				

MHLq-SVa—Mental Health Literacy Questionnaire; Mdn - Median; IQR - Interquartile range (P75-P25); U - Mann-Whitney test statistic; Z - Standardized Mann-Whitney test statistic; r - Effect size; H - Kruskal-Wallis's test statistic; df - Degrees of freedom; $\eta 2$ - Effect size.
